# Relationship Between Dietary Patterns and Chronic Diseases in Rural Population: Management Plays an Important Role in the Link

**DOI:** 10.3389/fnut.2022.866400

**Published:** 2022-04-13

**Authors:** Tiantian Li, Lizheng Guan, Xuan Wang, Xiaoying Li, Cui Zhou, Xianyun Wang, Wannian Liang, Rong Xiao, Yuandi Xi

**Affiliations:** ^1^Beijing Key Laboratory of Environmental Toxicology, School of Public Health, Capital Medical University, Beijing, China; ^2^Department of Geriatics, Beijing Jishuitan Hospital, Beijing, China; ^3^Vanke School of Public Health, Tsinghua University, Beijing, China

**Keywords:** chronic diseases, dietary pattern, health management, middle-aged and elderly people, rural population

## Abstract

**Objective:**

Health dietary pattern is related with reduced risk of chronic metabolic disease, but the benefits were not fully clear in the Chinese population. The aim of this study was to explore the association between dietary patterns and multiple chronic metabolic diseases in middle-aged and elderly Chinese.

**Methods:**

A total of 718 Chinese adults aged ≥ 45 who lived in the Huairou regions of Beijing were included in the present cross-sectional analysis from 2019 to 2020. Dietary data were obtained by food frequency questionnaires (FFQs). Dietary patterns were identified by principal components analysis (PCA). Logistic regression analysis and hierarchical analysis were used to examine the relationship among dietary patterns, health management, and chronic diseases.

**Results:**

Five dietary patterns were discovered in the subjects. The pattern with the higher percentage of energy supply by lipid was a risk factor for hypertension [odds ratio (OR) = 2.067, *p* = 0.013]. Lower energy intake (OR = 0.512, *p* = 0.012) and a reasonable ratio of dietary energy supply (OR = 0.506, *p* = 0.011) were beneficial to diabetes. The substitution of potato for grain might be an effective way of reducing diabetes (OR = 0.372, *p* < 0.001). The higher intake of high-quality protein was the protective factor for coronary heart disease (CHD; OR = 0.438, *p* = 0.008). Moderate intervention (OR = 0.185, *p* = 0.033) and appropriate health education (OR = 0.432, *p* = 0.016) could greatly subserve the prevention of chronic diseases, especially for hyperlipidemia. Men were more likely to be affected by health education, intervention, and follow-up than women. The prevalence of multimorbidity was higher in women (43.2%) than men (41.5%). The staple food intake and health management were also important factors to prevent multimorbidity.

**Conclusion:**

Dietary pattern with appropriate energy intake, a reasonable source of energy supply, high quality of macronutrients, and moderate management was associated with decreased risk of chronic metabolic diseases. Further studies are needed to clarify the cause–effect relationship between dietary patterns, health management, and chronic diseases and give suggestions to chronic metabolic disease prevention in middle-aged and elderly people in a rural area.

## Introduction

Dramatic gains in life expectancy have been made globally in the last couple of decades (1). However, the aging population leads to a growing burden of chronic diseases (2). Healthy aging, defined as increased healthy active years of life, may benefit from good habits, such as reasonable nutrition (3). The dietary pattern has been shown to be a major underlying factor that affects the health of elderly people (4). In that, a balanced diet is recognized as an effective way of reducing the risk of chronic metabolic diseases. The potential value of dietary pattern analysis is developing and evaluating food-based dietary guidelines, and it has emerged as an alternative and complementary approach for finding the association between diet and chronic diseases (5–8). Dietary pattern identification stresses the importance of holistic evaluation, indicating that different foods are consumed in intricacy combinations. Therefore, their synergistic effect on health should be considered when the diet-disease relationship was evaluated (9).

Nutritional transition, from higher intake of plant food to various dietary patterns, is a popular situation in Chinese. There is no doubt that dramatic change in dietary patterns and lifestyle in the last 20–30 years is an important reason for higher risk of chronic diseases (10, 11). With the increase of residents' income, the risk of chronic diseases among the elderly in Chinese rural areas continues to rise, which surpassed the prevalence of chronic diseases in urban areas for the first time in 2018 (12). According to our previous study, the elderly people in rural areas have become the focus population for changing dietary patterns and preventing chronic metabolic diseases, especially in Beijing of China (12, 13).

Different from the traditional research of single nutrients and foods (14), dietary pattern analysis was proposed as a “whole” nutritional assessment on different health outcomes (4, 15). A stronger evidence showed that dietary pattern analysis is better than individual nutrient and food when discussed the relationship between nutrition and chronic diseases (16). Huairou District is China's national demonstration area for comprehensive prevention and control of chronic diseases. The local government has taken great measures in health management. Considering its impact on chronic diseases, we also included some management factors into the statistical scope. In the present study, a cross-sectional study was conducted to find the associations between different dietary patterns and common chronic metabolic diseases in rural middle-aged and elderly people from Huairou of Beijing.

## Methods

### Study Design and Participants

In this study, rural population aged ≥45 was collected in Huairou of Beijing from 2019 to 2020 (subjects were derived from the baseline data conducted before the intervention study, registered at Chinese Clinical Trial Registry as ChiCTR2100054969). A multistage, stratified, cluster-sampling method was conducted, according to the geographical location, history, ethnicity, population distribution, etc. People with mental illness or who cannot complete questionnaires independently will be excluded. Finally, 718 individuals were included.

### Demographic and Measurements

The structured questionnaire was used to collect the data of demographics of participants that included age, sex, education level, income, and marital status. Clinical characteristics of subjects were collected by a self-guided questionnaire that includes hypertension, diabetes, hyperlipidemia, coronary heart disease (CHD), and stroke. The information of health management, such as community doctor follow-up (frequency categories: never, 1–3, 4–6, 7–9, 10–12, and above 12 times/year), village doctor intervention (frequency categories: never, 1–3, 4–6, 7–9, 10–12, and above 12 times/year), and village doctor health education (frequency categories: never, 1–3, 4–6, 7–10, and above 10 times/year), was detected. In addition, body mass index (BMI) was calculated by the formula of weight (kg)/height (m^2^).

### Dietary Assessment

A semi-quantitative food frequency questionnaire (FFQ) was used to do the dietary assessment. FFQ, which reflects the long-term dietary intake, is usually used to evaluate the dietary pattern of subjects (17, 18). There were five frequency categories in FFQ, which are as follows: (1) almost never eat or drink; (2) how many times per day; (3) how many times per week; (4) how many times per month; and (5) how many times per year. Standard portion size (e.g., tablespoon, bowl, and ladle) was recorded with the visual aids. Then, the frequency of each food item was converted into the daily intake. The China Food Composition Database was used to compute the daily energy consumption of participants. According to the Chinese Dietary Guidelines, all the food items were classified into 11 food groups as a criterion. Because sauces have been proven as one of main contributors to salt intake (19, 20), we converted sauce intake that includes soy sauce and sauce to salt intake through sodium, merged as “equivalent salt” with table salt. Finally, for each category of food, the total consumption was summing up all daily intake of every item in its classification.

### Statistical Analysis

Data were described as n (%) for categorical variables or median (interquartile range [IQR]) for continuous variables. Principal components analysis (PCA) was used to identify major dietary patterns. Component scores were obtained for the dietary pattern of every subject. The chi-square test was used for categorical variables analysis, and the rank-sum test was used to deal with non-normal distributions of continuous variables. The Kruskal–Wallis test was used to determine whether there were statistically significant differences in food-derived or nutrient intake between different patterns, and *post-hoc* comparisons were performed using the Dunn-Bonferroni method. The factors were rotated with an orthogonal rotation (varimax) to increase the interpretation and simplify the structure (21, 22). For each participant, factor scores were generated by multiple regression for every component, which was used in the final analysis, and factor loadings were based on the dietary intake. Quartile was classified based on the distribution in the whole population across the score of each dietary pattern that is used to describe the features of each pattern, build a regression model, and so on (17). The effects of basic characteristic and disease status on component scores of each dietary pattern were analyzed by a multivariate linear regression model. Model 1 was adjusted by individual characteristics (sex, age, education, marital status, nation, BMI, and income). The factors of health management (community doctor follow-up, village doctor intervention, and village doctor health education) were added in Model 2 based on adjusting Model 1. All statistical tests were two-sided, and the significance level was set at *p* ≤ 0.05. Statistical analyses were performed by IBM SPSS Statistics 26 and the R studio software program.

## Results

### Demographic Characteristics of Participants

Table 1 shows the demographic and clinical characteristics of all subjects. Participants (n = 718) had a mean (SD) age of 63.81 (8.71) years; 64.9% were women. In addition, the adult of middle-aged (45–64 years), young-old (65–79 years), and the old-old elderly (≥80 years) were 375 (52.2%), 316 (44.0%), 27 (3.8%) separately. The prevalence of each chronic diseases was 79.0% (hypertension), 27.9% (diabetes), 27.9% (hyperlipidemia), 19.4% (CHD), and 9.1% (stroke).

**Table 1:** Demographic features and distribution.

	**Total (*n* = 718)**	**Hypertension (*****n*** **=** **567)**			**Diabetes (*****n*** **=** **200)**			**Hyperlipidemia (*****n*** **=** **157)**			**Coronary heart disease (*****n*** **=** **139)**			**Stroke (*****n*** **=** **65)**		
		**Yes**	**No**	***p***	**Yes**	**No**	***p***	**Yes**	**No**	***p***	**Yes**	**No**	***p***	**Yes**	**No**	***p***
Gender, male, *n* (%)	252 (35.1)	186 (73.8)	66 (26.2)	0.013^*^	57 (22.6)	195 (77.4)^b^	0.021^*^	48 (19.0)	204 (81.0)	0.179	55 (21.8)	197 (78.2)	0.219	34 (13.5)	218 (86.5)	0.002^**^
**Age**, ***n*** **(%)**																
45~	375 (52.2)	288 (76.8)	87 (23.2)	0.289	114 (30.4)	261 (69.6)	0.131	96 (25.6)	279 (74.4)	0.016^*^	52 (13.9)^c^	323 (86.1)	<0.001^**^	24 (6.4)^d^	351 (93.6)	0.030^*^
65~	316 (44.0)	256 (81.0)	60 (19.0)		82 (25.9)	234 (74.1)		59 (18.7)	257 (81.3)		81 (25.6)^c^	235 (74.4)		37 (11.7)^d^	279 (88.3)	
80~	27 (3.8)	23 (85.2)	4 (14.8)		4 (14.8)	23 (85.2)		2 (7.4)	25 (92.6)		6 (22.2)	21 (77.8)		4 (14.8)	23 (85.2)	
**BMI**, ***n*** **(%)**																
<18.5	17 (2.4)	10 (58.8)^a^	7 (41.2)	0.043^*^	5 (29.4)	12 (70.6)	0.717	2 (11.8)	15 (88.2)	0.491	6 (35.3)	11 (64.7)	0.013^*^	2 (11.8)	15 (88.2)	0.577
18.5~	227 (31.6)	179 (78.9)	48 (21.1)		57 (25.1)	170 (74.9)		51 (22.5)	176 (77.5)		56 (24.7)^c^	171 (75.3)		22 (9.7)	205 (90.3)	
24~	287 (40.0)	221 (77.0)	66 (23.0)		85 (29.6)	202 (70.4)		58 (20.2)	229 (79.8)		51 (17.8)	236 (82.2)		28 (9.8)	259 (90.2)	
≥28	186 (25.9)	157 (84.4)^a^	29 (15.6)		53 (28.5)	133 (71.5)		46 (24.7)	140 (75.3)		26 (14.0)^c^	160 (86.0)		12 (6.5)	174 (93.5)	
**Nation**, ***n*** **(%)**																
Han	670 (93.3)	528 (78.8)	142 (21.2)	0.688	189 (28.2)	481 (71.8)	0.429	144 (21.5)	526 (78.5)	0.365	132 (19.7)	538 (80.3)	0.386	59 (8.8)	611 (91.2)	0.389
Manchu	48 (6.7)	39 (81.2)	9 (18.8)		11 (22.9)	37 (77.1)		13 (27.1)	35 (72.9)		7 (14.6)	41 (85.4)		6 (12.5)	42 (87.5)	
**Education**, ***n*** **(%)**																
Illiterate	108 (15.0)	89 (82.4)	19 (17.6)	0.284	33 (30.6)	75 (69.4)	0.318	21 (19.4)	87 (80.6)	0.887	21 (19.4)	87 (80.6)	0.045^*^	10 (9.3)	98 (90.7)	0.297
Primary school	230 (32.0)	186 (80.9)	44 (19.1)		57 (24.8)	173 (75.2)		49 (21.3)	181 (78.7)		59 (25.7)^c^	171 (74.3)		27 (11.7)	203 (88.3)	
Junior high school	262 (36.5)	204 (77.9)	58 (22.1)		71 (27.1)	191 (72.9)		58 (22.1)	204 (77.9)		39 (14.9)^c^	223 (85.1)		21 (8.0)	241 (92.0)	
High school	103 (14.3)	79 (76.7)	24 (23.3)		32 (31.1)	71 (68.9)		26 (25.2)	77 (74.8)		17 (16.5)	86 (83.5)		5 (4.9)	98 (95.1)	
Junior college	15 (2.1)	9 (60.0)	6 (40.0)		7 (46.7)	8 (53.3)		3 (20.0)	12 (80.0)		3 (20.0)	12 (80.0)		2 (13.3)	13 (86.7)	
**Marital status**, ***n*** **(%)**																
Single	20 (2.8)	16 (80.0)	4 (20.0)	0.074	2 (10.0)	18 (90.0)	0.323	2 (10.0)	18 (90.0)	0.211	3 (15.0)	17 (85.0)	<0.001^**^	3 (15.0)	17 (85.0)	0.042^*^
Married	616 (85.8)	492 (79.9)	124 (20.1)		173 (28.1)	443 (71.9)		142 (23.1)	474 (76.9)		108 (17.5)^c^	508 (82.5)		51 (8.3) ^d^	565 (91.7)	
Separated/divorced	9 (1.3)	4 (44.4)	5 (55.6)		3 (33.3)	6 (66.7)		2 (22.2)	7 (77.8)		0 (0.0)	9 (100.0)		3 (33.3) ^d^	6 (66.7)	
Widower	70 (9.7)	54 (77.1)	16 (22.9)		21 (20.0)	49 (70.0)		10 (14.3)	60 (85.7)		27 (38.6)^c^	43 (61.4)		8 (11.4)	62 (88.6)	
**Income**, ***n*** **(%)**																
<10,000	164 (22.8)	127 (77.4)	37 (22.6)	0.743	48 (29.3)	116 (70.7)	0.826	32 (19.5)	132 (80.5)	0.092	39 (23.8)	125 (76.2)	0.081	18 (11.0)	146 (89.0)	0.068
10,000~	311 (43.3)	245 (78.8)	66 (21.2)		82 (26.4)	229 (73.6)		62 (19.9)	249 (80.1)		65 (20.9)	246 (79.1)		36 (11.6)	275 (88.4)	
30,000~	119 (16.6)	93 (78.2)	26 (21.8)		34 (28.6)	85 (71.4)		38 (31.9)	81 (68.1)		15 (12.6)	104 (87.4)		6 (5.0)	113 (95.0)	
50,000~	64 (8.9)	55 (85.9)	9 (14.1)		20 (31.2)	44 (68.8)		15 (23.4)	49 (76.6)		12 (18.8)	52 (81.2)		3 (4.7)	61 (95.3)	
70,000~	28 (3.9)	21 (75.0)	7 (25.0)		10 (35.7)	18 (64.3)		7 (25.0)	21 (75.0)		7 (25.0)	21 (75.0)		1 (3.6)	27 (96.4)	
90,000~	23 (3.2)	17 (73.9)	6 (26.1)		5 (21.7)	18 (78.3)		3 (13.0)	20 (87.0)		1 (4.3)	22 (95.7)		0 (0.0)	23 (100.0)	
**Community doctor follow-up**, ***n*** **(%)**																
Never	289 (40.3)	228 (40.2)	61 (40.4)	0.653	84 (42)	205 (39.6)	0.125	71 (45.2)	218 (38.9)	0.455	62 (44.6)	227 (39.2)	0.490	23 (35.4)	266 (40.7)	0.741
1–3 times/year	239 (33.3)	188 (33.2)	51 (33.8)		57 (28.5)	182 (35.1)		52 (33.1)	187 (33.3)		43 (30.9)	196 (33.9)		25 (38.5)	214 (32.8)	
4–6 times/year	103 (14.3)	85 (15)	18 (11.9)		39 (19.5)	64 (12.4)		20 (12.7)	83 (14.8)		14 (10.1)	89 (15.4)		9 (13.8)	94 (14.4)	
7–9 times/year	26 (3.6)	18 (3.2)	8 (5.3)		7 (3.5)	19 (3.7)		6 (3.8)	20 (3.6)		5 (3.6)	21 (3.6)		4 (6.2)	22 (3.4)	
10–12 times/year	31 (4.3)	26 (4.6)	5 (3.3)		7 (3.5)	24 (4.6)		5 (3.2)	26 (4.6)		8 (5.8)	23 (4.0)		2 (3.1)	29 (4.4)	
Above 12 times/year	30 (4.2)	22 (3.9)	8 (5.3)		6 (3)	24 (4.6)		3 (1.9)	27 (4.8)		7 (5.0)	23 (4.0)		2 (3.1)	28 (4.3)	
**Village doctor Intervention**, ***n*** **(%)**																
Never	316 (44.0)	243 (42.9)	73 (48.3)	0.873	90 (45.0)	226 (43.6)	0.832	71 (45.2)	245 (43.7)	0.222	72 (51.8)	244 (42.1)	0.197	33 (50.8)	283 (43.3)	0.571
1–3 times/year	174 (24.2)	139 (24.5)	35 (23.2)		45 (22.5)	129 (24.9)		39 (24.8)	135 (24.1)		26 (18.7)	148 (25.6)		16 (24.6)	158 (24.2)	
4–6 times/year	96 (13.4)	77 (13.6)	19 (12.6)		31 (15.5)	65 (12.5)		23 (14.6)	73 (13.0)		16 (11.5)	80 (13.8)		6 (9.2)	90 (13.8)	
7–9 times/year	24 (3.3)	19 (3.4)	5 (3.3)		7 (3.5)	17 (3.3)		7 (4.5)	17 (3.0)		2 (1.4)	22 (3.8)		3 (4.6)	21 (3.2)	
10–12 times/year	37 (5.2)	31 (5.5)	6 (4)		8 (4.0)	29 (5.6)		2 (1.3)	35 (6.2)		7 (5.0)	30 (5.2)		1 (1.5)	36 (5.5)	
Above 12 times/year	71 (9.9)	58 (10.2)	13 (8.6)		19 (9.5)	52 (10.0)		15 (9.6)	56 (10.0)		16 (11.5)	55 (9.5)		6 (9.2)	65 (10.0)	
**Village doctor health education**, ***n*** **(%)**																
Never	115 (16.0)	88 (17.9)	27 (15.5)	0.635	37 (18.5)	78 (15.1)	0.464	34 (21.7)	81 (14.4)	0.255	23 (16.5)	92 (15.9)	0.968	10 (15.4)	105 (16.1)	0.936
1–3 times/year	308 (42.9)	240 (45.0)	68 (42.3)		85 (42.5)	223 (43.1)		63 (40.1)	245 (43.7)		59 (42.4)	249 (43.0)		30 (46.2)	278 (42.6)	
4–6 times/year	145 (20.2)	118 (17.9)	27 (20.8)		35 (17.5)	110 (21.2)		27 (17.2)	118 (21)		26 (18.7)	119 (20.6)		14 (21.5)	131 (20.1)	
7–10 times/year	38 (5.3)	33 (3.3)	5 (5.8)		8 (4)	30 (5.8)		9 (5.7)	29 (5.2)		7 (5.0)	31 (5.4)		3 (4.6)	35 (5.4)	
Above 10 times/year	112 (15.6)	88 (15.9)	24 (15.5)		35 (17.5)	77 (14.9)		24 (15.3)	88 (15.7)		24 (17.3)	88 (15.2)		8 (12.3)	104 (15.9)	

* *p < 0.05*,

** *p < 0.01*.

a *p < 0.05; difference between two proportions in hypertension*.

b *p < 0.05; difference between two proportions in diabetes*.

c *p < 0.05; difference between two proportions in coronary heart disease*.

d *p < 0.05; difference between two proportions in stroke*.

As shown in Table 1, a significant higher prevalence of hypertension, diabetes, and stroke in women is found when compared with men. Young-old people (65–79 years) were more likely to be CHD and stroke. In addition, obese people had a higher probability to suffer from hypertension and CHD. Poorly educated people were at higher risk of CHD.

### Comparison of Dietary Intake in Different Chronic Diseases

As shown in Table 2, people with hypertension are more likely to have a higher intake of vegetables and fruits. Diabetics ingested more milk and dairy products, while having lower consumption of nuts, fruits, sugar, and alcohol. Hyperlipidemia patients consumed more seafood. Subjects of CHD were liable to intake less meat and grease. These results made us focus on the dietary pattern analysis in the next step.

**Table 2:** Dietary intake of the study population across chronic diseases categories.

	**Total**	**Hypertension**			**Diabetes**			**Hyperlipidemia**			**Coronary heart disease**			**Stroke**		
		**Yes**	**No**	***p***	**Yes**	**No**	***p***	**Yes**	**No**	***p***	**Yes**	**No**	***p***	**Yes**	**No**	***p***
Cereals and tubers	307.3 (213.7, 406.5)	312.8 (215.7, 403.3)	300.3 (207.1, 410.0)	0.454	289.3 (202.4, 391.1)	318.4 (215.9, 414.3)	0.080	289.6 (205.5, 411.7)	314.3 (215.0, 411.7)	0.200	303.1 (203.3, 413.8)	312.9 (214.3, 403.6)	0.766	335.7 (220.8, 433.0)	306.5 (213.6, 400.6)	0.384
Cereals	287.1 (200.0, 385.2)	291.4 (200.0, 385.2)	275.6 (185.7, 385.7)	0.366	278.7 (187.6, 371.7)	292.9 (200.8, 387.4)	0.204	278.6 (185.9, 361.9)	291.4 (200.0, 385.7)	0.293	278.2 (185.7, 385.9)	289.1 (200.0, 379.4)	0.706	300.0 (196.2, 408.8)	286.5 (200.0, 377.8)	0.534
Tubers	6.7 (0.0, 28.6)	6.7 (0.0, 28.6)	10.0 (0.0, 28.6)	0.428	6.7 (0.0, 28.6)	7.1 (0.0, 28.6)	0.101	10.0 (0.0, 23.2)	6.7 (0.0, 28.6)	0.892	7.1 (0.0, 28.6)	6.7 (0.0, 28.6)	0.809	14.3 (0.0, 35.7)	6.7 (0.0, 28.6)	0.796
Meat	36.4 (10.0, 72.8)	38.1 (11.3, 72.9)	28.8 (8.3, 71.4)	0.384	37.5 (11.4, 72.7)	35.8 (9.9, 72.8)	1.000	44.8 (14.3, 93.0)	35.7 (8.8, 71.4)	0.084	23.4 (5.7, 56.7)	42.9 (13.3, 78.6)	0.001^**^	42.9 (14.6, 87.9)	35.7 (9.8, 72.2)	0.492
Soy and nuts	26.7 (7.1, 70.3)	29.6 (7.5, 73.6)	23.2 (6.7, 59.3)	0.125	22.2 (5.9, 59.9)	29.5 (7.6, 73.7)	0.057	27.2 (10.0, 72.6)	26.6 (6.6, 69.8)	0.681	27.5 (7.0, 83.0)	26.6 (7.1, 66.0)	0.480	30.6 (9.1, 59.6)	26.5 (6.9, 71.2)	0.787
Soybeans	6.9 (2.3, 16.7)	6.9 (2.3, 16.9)	6.6 (2.3, 16.5)	0.667	7.3 (2.2, 16.5)	6.8 (2.2, 17.2)	0.945	8.3 (2.1, 16.0)	6.6 (2.3, 17.4)	0.655	8.3 (3.3, 16.8)	6.7 (2.2, 16.5)	0.232	8.3 (3.3, 22.3)	6.7 (2.3, 16.5)	0.227
Nuts	10.7 (0.0, 51.0)	11.7 (0.0, 55.0)	8.6 (0.0, 31.3)	0.370	7.0 (0.0, 42.9)	14.3 (0.0, 55.9)	0.025^*^	7.1 (0.0, 50.7)	11.4 (0.0, 51.0)	0.787	8.8 (0.0, 67.1)	11.3 (0.0, 50.0)	0.759	10.7 (0.0, 46.4)	10.7 (0.0, 52.6)	0.598
Vegetables	210.0 (121.4, 350.0)	214.3 (129.1, 360.7)	200.0 (106.7, 313.7)	0.026^*^	207.1 (119.8, 392.1)	210.6 (122.8, 342.9)	0.949	225.0 (118.6, 423.3)	207.4 (124.1, 339.2)	0.548	218.9 (114.3, 328.6)	209.7 (128.3, 353.1)	0.652	253.7 (100.0, 347.5)	207.6 (128.6, 350.6)	0.947
Fruits	100.0 (16.8, 200.0)	100.0 (21.4, 200.0)	57.1 (6.7, 200.0)	0.013^*^	57.1 (6.7, 200.0)	100.0 (26.8, 200.0)	0.001^**^	100.0 (28.6, 200.0)	85.7 (14.3, 200.0)	0.353	100.0 (20.0, 200.0)	92.4 (16.7, 200.0)	0.791	71.4 (22.5, 200.0)	100.0 (16.7, 200.0)	0.920
Seafood	0.8 (0.0, 6.7)	0.9 (0.0, 6.7)	0.6 (0.0, 6.7)	0.827	1.3 (0.0, 6.7)	0.8 (0.0, 6.7)	0.370	1.7 (0.0, 8.3)	0.8 (0.0, 6.7)	0.039^*^	1.3 (0.0, 5.0)	0.8 (0.0, 6.9)	0.520	0.3 (0.0, 3.3)	0.8 (0.0, 6.7)	0.100
Milk and dairy products	35.7 (0.0, 250.0)	35.7 (0.0, 250.0)	0.6 (0.0, 250.0)	0.090	71.4 (0.0, 250.0)	33.3 (0.0, 250.0)	0.004^**^	57.1 (0.0, 250.0)	35.7 (0.0, 250.0)	0.231	50.0 (0.0, 250.0)	35.7 (0.0, 250.0)	0.656	64.3 (0.0, 250.0)	35.7 (0.0, 250.0)	0.206
Eggs	50.0 (14.3, 50.0)	50.0 (14.3, 50.0)	50.0 (14.3, 50.0)	0.983	50.0 (14.3, 50.0)	50.0 (14.3, 50.0)	0.946	50.0 (9.3, 50.0)	50.0 (14.3, 50.0)	0.055	50.0 (14.3, 50.0)	50.0 (14.3, 50.0)	0.588	50.0 (14.3, 50.0)	50.0 (14.2, 50.0)	0.875
Grease	33.3 (16.7, 50.0)	30.6 (16.7, 50.0)	33.3 (13.9, 55.5)	0.928	33.3 (13.9, 52.5)	33.3 (16.7, 50.0)	0.540	27.8 (13.9, 51.7)	33.3 (16.7, 50.0)	0.281	27.8 (13.9, 43.3)	33.3 (16.7, 53.3)	0.044^*^	27.8 (13.9, 66.7)	33.3 (16.7, 50.0)	0.732
Salt	6.7 (4.0, 10.0)	6.7 (3.9, 10.0)	6.7 (4.2, 8.3)	0.770	6.7 (3.3, 10.0)	6.7 (4.1, 10.0)	0.811	6.7 (3.3, 11.7)	6.7 (4.2, 10.0)	0.873	8.3 (4.2, 11.1)	6.7 (3.9, 10.0)	0.187	8.3 (4.2, 13.3)	6.7 (3.9, 10.0)	0.130
Sugar	0.0 (0.0, 1.7)	0.0 (0.0, 2.1)	0.0 (0.0, 1.7)	0.145	0.0 (0.0, 0.1)	0.4 (0.0, 3.3)	<0.001^**^	0.0 (0.0, 1.6)	0.0 (0.0, 2.1)	0.718	0.0 (0.0, 1.7)	0.0 (0.0, 2.1)	0.770	0.2 (0.0, 2.8)	0.0 (0.0, 1.7)	0.333
Equivalent salt	8.8 (5.3, 12.6)	8.7 (5.4, 12.9)	8.9 (5.0, 12.0)	0.505	8.5 (4.8, 12.5)	8.9 (5.5, 12.7)	0.550	8.7 (4.6, 13.6)	8.8 (5.4, 12.3)	0.871	9.2 (5.7, 13.3)	8.7 (5.2, 12.5)	0.419	9.6 (6.1, 16.3)	8.7 (5.1, 12.5)	0.161
Alcohol	0.0 (0.0, 0.6)	0.0 (0.0, 0.8)	0.0 (0.0, 0.4)	0.988	0.0 (0.0, 0.0)	0.0 (0.0, 7.1)	0.024^*^	0.0 (0.0, 0.3)	0.0 (0.0, 1.3)	0.630	0.0 (0.0, 7.1)	0.0 (0.0, 7.1)	0.534	0.0 (0.0, 31.0)	0.0 (0.0, 0.4)	0.257

* *p < 0.05*,

** *p < 0.01*.

### Dietary Pattern

Factor analysis revealed five main dietary patterns, which together explained 49.3% of the total variance in dietary intake. An eigenvalue cutoff >1, scree plot, and component interpretability were used to decide the number of components to retain. We found a significant χ^2^ (*p* < 0.001) for Bartlett's test of sphericity and the Kaiser–Meyer–Olkin test >0.6, indicating that the correlation among the variables was strong enough for factor analysis.

The characteristics of five dietary patterns are shown in Tables 3, 4. Food groups with an absolute factor loading coefficient of 0.5 and above were strongly correlated within the pattern. Pattern 1, which explained 13.8% of the total variance, was characterized by the consumption of cereals and vegetables. However, it showed the highest energy intake and higher percentage of energy from carbohydrates in all five patterns. People in pattern 2, which explained 10.2%, were more likely to eat meat and seafood. While, it was the one that the percentages of energy supplied by carbohydrate (PEC), protein (PEP), and fat (PEF) were all in the range of Chinese Dietary Reference Intakes. Pattern 3, explained 8.8%, had higher consumption of fruits, milk, and dairy products. This pattern contained more animal food-derived protein than others, which was recognized as high-quality protein. Pattern 4, explained 8.5%, included grease, sugar, and equivalent salt. The total energy in this pattern was lower than others, but the PEF was the highest one. The characteristic of pattern 5 (explained 8.0%) was that tubers were the main sources of carbohydrate instead of grain and the energy intake was the lowest one.

**Table 3:** Factor-loading matrix for the dietary patterns and food groups.

	**Pattern 1**	**Pattern 2**	**Pattern 3**	**Pattern 4**	**Pattern 5**
Cereals	0.693^*^	0.160	−0.246	−0.095	0.068
Tubers	0.147	0.086	0.112	−0.023	0.685^*^
Meat	0.348	0.535^*^	0.097	0.075	−0.083
Soybeans	0.396	0.157	0.167	0.047	−0.424
Nuts	0.392	0.290	0.362	0.018	0.174
Vegetables	0.670^*^	−0.197	0.153	0.062	0.021
Fruits	0.067	0.099	0.612^*^	−0.011	0.050
Seafoods	−0.130	0.746^*^	−0.018	−0.074	0.075
Milk and dairy products	−0.038	−0.038	0.730^*^	−0.051	−0.110
Eggs	0.049	0.375	0.170	0.117	−0.416
Grease	−0.062	−0.232	0.118	0.733^*^	−0.062
Sugar	0.029	0.225	0.009	0.554^*^	0.446
Equivalent salt	0.073	0.162	−0.301	0.613^*^	−0.172

* *Means factor loading with absolute value ≥ 0.5*.

**Table 4:** Different food-derived and nutrients intake among the highest quartiles of five patterns.

	**Pattern 1**	**Pattern 2**	**Pattern 3**	**Pattern 4**	**Pattern 5**	***p***
Carbohydrate (g/d)	455.5 (364.0, 553.5)	370.6 (289.0, 483.4)	345.2 (277.2, 446.6)^a^^b^	302.7 (221.1, 399.6)^a^^b^^c^	358.0 (280.8, 466.8)^a^^b^^c^^d^	<0.001^**^
Animal food-derived (%)	1.7 (0.6, 8.6)	4.2 (1.3, 13.8)^a^	17.7 (8.4, 26.8)^a^^b^	3.4 (0.8, 13.2)^c^	1.5 (0.5, 10.5)^b^^c^	<0.001^**^
Bean-derived (%)	3.4 (0.9, 9.2)	3.1 (0.9, 5.7)	2.4 (0.7, 5.5)	2.6 (0.7, 6.5)	1.5 (0.3, 4.6)^a^^b^^d^	<0.001^**^
Grain-derived (%)	76.4 (66.6, 84.5)	75.4 (64.9, 83.5)	58.7 (49.9, 65.9)^a^^b^	75.3 (65.4, 84.5)^c^	79.5 (67.8, 88.0)^c^	<0.001^**^
Other food-derived (%)	12.2 (7.6, 18.4)	12.2 (7.3, 18.7)	16.2 (11.6, 23.7)^a^^b^	12.1 (7.1, 18.5)^c^	11.9 (7.4, 18.3)^c^	<0.001^**^
Protein (g/d)	97.8 (75.8, 120.0)	91.7 (68.7, 114.3)^a^	88.2 (70.6, 107.5)^a^	63.3 (45.5, 89.2)^a^^b^^c^	68.5 (49.4, 92.2)^a^^b^^c^	<0.001^**^
Animal food-derived (%)	23.9 (14.1, 36.3)	37.9 (26.4, 49.2)^a^	44.7 (33.9, 58.1)^a^^b^	30.4 (17.2, 43.4)^a^^b^^c^	22.9 (11.9, 36.6)^b^^c^^d^	<0.001^**^
Bean-Derived (%)	4.8 (1.9, 13.3)	4.3 (1.9, 9.7)	4.4 (1.7, 8.5)	5.1 (2.2, 10.8)	2.4 (0.9, 5.1)^a^^b^^c^^d^	<0.001^**^
Grain-derived (%)	46.9 (35.0, 60.5)	38.4 (28.6, 50.6)^a^^c^	30.2 (22.9, 38.5)^a^^b^	43.5 (32.7, 54.3)^c^	51.7 (38.2, 70.5)^b^^c^^d^	<0.001^**^
Other food-derived (%)	14.4 (8.5, 23.2)	10.9 (6.1, 17.9)^a^^c^	14.8 (8.6, 21.9)	11.0 (7, 18.1)	12.9 (8.1, 24.8)	0.001^**^
Fat (g/d)	102.3 (70.6, 138.6)	101.3 (67.6, 140.5)^a^	116.3 (88.4, 150.7)^a^^b^	130.2 (98.0, 173.0)^a^^b^^c^	77.2 (48.5, 118.7)^a^^d^	<0.001^**^
Animal food-derived (%)	33.1 (18.9, 51.2)	46.0 (30.5, 59.0)^a^	41.7 (30.1, 54.0)^a^	20.7 (10.2, 32.8)^a^^b^^c^	27.5 (14.3, 41.5)^a^^b^^c^	<0.001^**^
Bean-Derived (%)	2.3 (0.8, 5.9)	1.9 (0.7, 4.6)	1.4 (0.5, 3.4)^a^	1.2 (0.5, 2.7)^a^^b^	1.0 (0.3, 2.0)^a^^b^	<0.001^**^
Grain-Derived (%)	6.7 (4.0, 10.8)	5.7 (3.6, 9.2)	3.6 (2.4, 5.2)^a^^b^	3.4 (2.1, 5.3)^a^^b^	8.5 (4.9, 13.8)^b^^c^^d^	<0.001^**^
Other food-derived (%)	52.2 (34.3, 67.3)	44.4 (29.1, 59.7)^a^	51.6 (36.1, 64.3)	72.8 (59.3, 83.4)^a^^b^^c^	61.2 (46.1, 75.2)^a^^b^^c^^d^	<0.001^**^
Energy intake (kcal)	3,012.6 (2,581.3, 3,556.5)	2,712.6 (2,052.5, 3,441.9)^a^	2,725.9 (2,215.5, 3,276.0)^a^	2,578.3 (2,033.1, 3,222.2)^a^^d^^c^	2,484.9 (1,820.2, 3,140.2)^a^^b^^c^^d^	<0.001^**^
**Energy source**						
Carbohydrate energy ratio (%E)	61.4 (53.4, 70.6)	56.7 (47.2, 64.7)^a^	51.9 (44.8, 58.3)^a^^b^	47.4 (38.7, 56.3)^a^^b^^c^	62.6 (54.3, 68.6)^b^^c^^d^	<0.001^**^
Protein energy ratio (%E)	12.7 (11.3, 14.1)	13.4 (12.4, 14.7)^a^	13.3 (11.7, 14.4)^a^^b^	10.6 (8.6, 12.6)^a^^b^^c^	11.5 (10.1, 13.2)^b^^c^^d^	<0.001^**^
Fat energy ratio (%E)	31.2 (22.3, 38.6)	34.0 (27.5, 43.0)^a^	39.6 (33.1, 46.0)^a^^b^	45.3 (36.6, 54.6)^a^^b^^c^	30.0 (22.6, 39.0)^a^^b^^c^^d^	<0.001^**^

*Statistically different with Pattern*

** *p < 0.01*.

a *p < 0.05; statistically different with Pattern 1*.

b *p < 0.05; statistically different with Pattern 2*.

c *p < 0.05; statistically different with Pattern 3*.

d *p < 0.05; statistically different with Pattern 4*.

### Association Between Demographic and Clinical Characteristics and Dietary Pattern

Results of the linear regression models for the relationship between demographic characteristics and food component scores could be found in Table 5. Sex and income (β_sex_ = −0.152, 95% CI −0.483, −0.155; β_income_ = 0.110, 95% CI 0.010, 0.068) were main influential factors in pattern 1. The factors affected pattern 2 were sex, age, BMI, and income (β_sex_ = −0.099, 95% CI −0.369, −0.045; β_age_ = −0.098, 95% CI −0.021, −0.002; β_BMI_ = −0.097, 95% CI −0.046, −0.006; β_income_ = 0.091, 95% CI 0.004, 0.061). Pattern 3 was associated with sex, age, education, and income (β_sex_ = 0.125, 95% CI 0.098, 0.428; β_age_ = 0.108, 95% CI 0.003, 0.022; β_education_ = 0.094, 95% CI 0.006, 0.188; β_income_ = 0.101, 95% CI 0.007, 0.065). None was found in patterns 4 and 5.

**Table 5:** Association between dietary patterns and characteristic.

	**Pattern 1**		**Pattern 2**		**Pattern 3**		**Pattern 4**		**Pattern 5**	
	**β**	***p***	**β**	***p***	**β**	***p***	**β**	***p***	**β**	***p***
Sex	−0.152	<0.001^**^	−0.099	0.012^*^	0.125	0.002^**^	−0.051	0.207	0.010	0.795
Age	−0.030	0.471	−0.098	0.019^*^	0.108	0.011^*^	−0.042	0.327	−0.011	0.789
BMI	0.028	0.464	−0.097	0.009^**^	0.011	0.769	0.018	0.644	−0.075	0.051
Nation	0.044	0.244	0.003	0.927	−0.001	0.984	0.021	0.572	0.011	0.770
Education	−0.067	0.137	0.076	0.086	0.094	0.037^*^	−0.028	0.539	−0.010	0.833
Income	0.110	0.008^**^	0.091	0.025^*^	0.101	0.015^*^	−0.069	0.099	0.040	0.342

* *p < 0.05*,

** *p < 0.01*.

Table 6 presents the results of multivariate linear regression models which showed the effectiveness of dietary patterns on each chronic disease after adjusting demographic characteristics. The relationships between hypertension and pattern 1 (β_pattern__1_ = 0.103, 95% CI 0.074, 0.432), diabetes and pattern 4 or pattern 5 (β_pattern4_ = −0.094, 95% CI −0.373, −0.044; β_pattern5_ = −0.142, 95% CI −0.481, −0.153) were found in present study.

**Table 6:** Association between dietary patterns and chronic diseases.

	**Pattern 1**		**Pattern 2**		**Pattern 3**		**Pattern 4**		**Pattern 5**	
	**β**	***p***	**β**	***p***	**β**	***p***	**β**	***p***	**β**	***p***
Hypertension^a^	0.103	0.006^**^	0.012	0.749	−0.030	0.426	0.012	0.753	0.019	0.620
Diabetes^a^	−0.038	0.304	−0.040	0.283	−0.038	0.311	−0.094	0.013^*^	−0.142	<0.001^**^
Hyperlipidemia^a^	−0.014	0.716	0.033	0.371	0.001	0.980	−0.036	0.345	−0.032	0.391
Coronary heart disease^a^	−0.014	0.719	−0.003	0.944	−0.039	0.313	0.018	0.645	0.018	0.648
Stroke^a^	0.022	0.562	−0.027	0.462	0.056	0.140	0.003	0.930	0.000	0.993

a *Means adjusted for sex, age, body mass index (BMI), nation, education, and income*.

* *p < 0.05*,

** *p < 0.01*.

### The Effect of Dietary Pattern on Chronic Diseases

**Figure 1:**
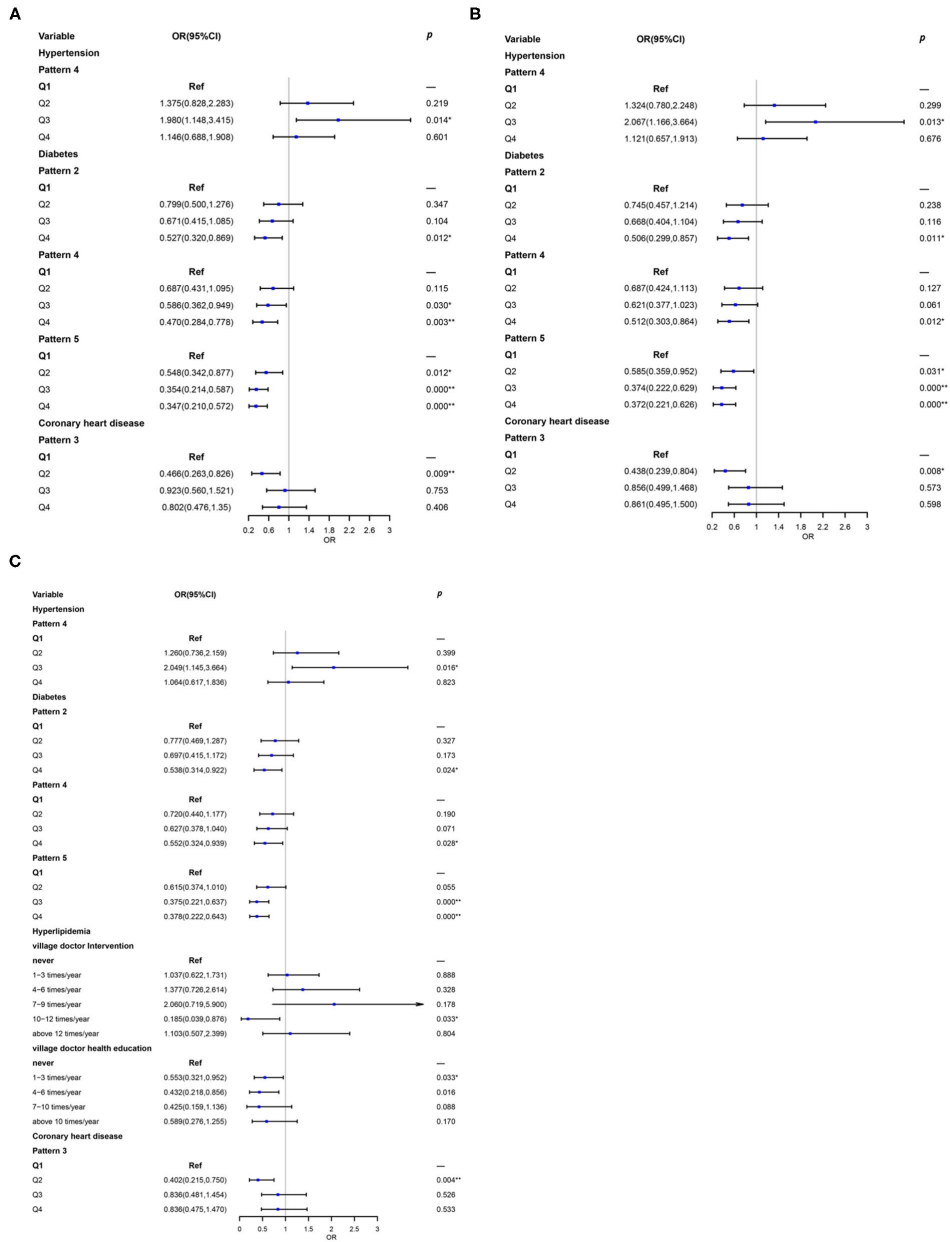
Association between dietary patterns and prevalent metabolic diseases **(A)** unadjusted; **(B)** Model 1: adjusted for sex, age, education, marital status, nation, body mass index (BMI), and income; **(C)** Model 2: add community doctor follow-up, village doctor intervention, and village doctor health education based on Model 1. Q1 was the reference group. *p < 0.05, **p < 0.01.

Results of logistic regression analysis were manifested in Figure 1. Compared with the reference group, Q3 of the pattern 4 was the independent risk factor for hypertension either in the crude model (OR 1.980, 95% CI 1.148, 3.415, *p* = 0.014) or in model 1 [odds ratio (OR) 2.067 95% CI 1.166, 3.664, *p* = 0.013]. It was shown that too much condiment intake and illogical PEF probably increased the prevalence of hypertension. It was noteworthy that diabetes was closely associated with the dietary patterns. Q4 of pattern 2 (OR 0.506, 95% CI 0.299, 0.587, *p* = 0.011), Q4 of pattern 4 (OR 0.512, 95% CI 0.303, 0.864, *p* = 0.012), and Q2 (OR 0.585, 95% CI 0.359, 0.952, *p* = 0.031), Q3 (OR 0.374, 95% CI 0.222, 0.629, *p* < 0.001), Q4 (OR 0.372, 95% CI 0.221, 0.626, *p* < 0.001) of pattern 5 were all the protective factors of diabetes in both models. In CHD, Q2 of pattern 3 was an important protective factor in the crude model (OR 0.466, 95% CI 0.263, 0.826, *p* = 0.009). Meanwhile, the extent of this protection was increased in model 1 (OR 0.438, 95% CI 0.239, 0.804, *p* = 0.008).

To observe the performance of different genders, these results stratified by sex were next explored (Table 7). After adjusting for age, education, marital status, nation, BMI, and income, Q3 of pattern 4 was definitely the risk factor for hypertension (OR 3.198, 95% CI 1.189, 8.602) in men and Q4 of pattern 1 was in women (OR 3.203, 95% CI 1.349, 7.606). Q4 of pattern 2 (OR 0.167, 95% CI 0.054, 0.511), and Q3 (OR 0.246, 95% CI 0.086, 0.706), Q4 (OR 0.255, 95% CI 0.087, 0.749) of pattern 5 were directly related to a lower prevalence of diabetes in men. Furthermore, Q3 (OR 0.384, 95% CI 0.204, 0.724) and Q4 (OR 0.411, 95% CI 0.219, 0.770) of pattern 5 were significantly associated with the lower prevalence of diabetes in women. Besides, pattern 3 was associated with a lower prevalence of CHD in women (OR 0.289, 95% CI 0.127, 0.658). What is more, pattern 1 was an independent risk factor for stroke in men (OR 12.38, 95% CI 1.777, 86.234).

**Table 7:** Factors contributing to different chronic diseases across sex categories.

	**Unadjusted**			**Model 1**			**Model 2**		
	**OR**	**95% CI**	***p***	**OR**	**95% CI**	***p***	**OR**	**95% CI**	***p***
**Male**									
**Hypertension**									
**Pattern 4**									
Q1	Ref	–	–	Ref	–	–	Ref	–	–
Q2	1.873	0.796, 4.407	0.150	2.081	0.783, 5.533	0.142	2.190	0.763, 6.285	0.145
Q3	2.792	1.152, 6.765	0.023^*^	3.198	1.189, 8.602	0.021^*^	3.754	1.287, 10.944	0.015^*^
Q4	1.203	0.544, 2.659	0.649	1.176	0.486, 2.842	0.720	1.118	0.438, 2.854	0.815
**Diabetes**									
**Pattern 2**									
Q1	Ref	–	–	Ref	–	–	Ref	–	–
Q2	1.034	0.424, 2.526	0.941	0.744	0.254, 2.183	0.590	0.665	0.207, 2.137	0.493
Q3	0.609	0.243, 1.526	0.290	0.583	0.199, 1.707	0.325	0.423	0.125, 1.432	0.167
Q4	0.308	0.120, 0.787	0.014^*^	0.167	0.054, 0.511	0.002^**^	0.160	0.047, 0.538	0.003^**^
**Pattern 5**									
Q1	Ref	–	–	Ref	–	–	Ref	–	–
Q2	0.523	0.210, 1.304	0.164	0.406	0.141, 1.165	0.094	0.563	0.179, 1.771	0.326
Q3	0.363	0.141, 0.934	0.035^*^	0.246	0.086, 0.706	0.009^**^	0.316	0.100, 0.999	0.050
Q4	0.295	0.109, 0.796	0.016^*^	0.255	0.087, 0.749	0.013^*^	0.370	0.111, 1.238	0.107
**Village doctor health education**									
Never	–	–	–	–	–	–	Ref	–	–
1–3 times/year	–	–	–	–	–	–	0.866	0.271, 2.769	0.808
4–6 times/year	–	–	–	–	–	–	0.196	0.041, 0.953	0.043^*^
7–10 times/year	–	–	–	–	–	–	0.174	0.019, 1.637	0.126
Above 10 times/year	–	–	–	–	–	–	0.232	0.046, 1.171	0.077
**Hyperlipidemia**									
**Pattern 5**									
Q1	Ref	–	–	Ref	–	–	Ref	–	–
Q2	1.111	0.373, 3.305	0.850	1.556	0.462, 5.239	0.475	1.538	0.411, 5.754	0.523
Q3	1.742	0.607, 4.998	0.302	2.146	0.642, 7.171	0.215	1.915	0.515, 7.123	0.332
Q4	2.340	0.886, 6.180	0.086	2.809	0.943, 8.370	0.064	4.398	1.235, 15.660	0.022^*^
**Village doctor health education**									
Never	–	–	–	–	–	–	Ref	–	–
1–3 times/year	–	–	–	–	–	–	0.382	0.091, 1.594	0.187
4–6 times/year	–	–	–	–	–	–	0.251	0.053, 1.201	0.083
7–10 times/year	–	–	–	–	–	–	0.050	0.005, 0.517	0.012^*^
Above 10 times/year	–	–	–	–	–	–	0.592	0.118, 2.973	0.525
**CHD**									
**Community doctor follow-up**									
Never	–	–	–	–	–	–	Ref	–	–
1–3 times/year	–	–	–	–	–	–	0.320	0.118, 0.867	0.025^*^
4–6 times/year	–	–	–	–	–	–	0.488	0.126, 1.888	0.298
7–9 times/year	–	–	–	–	–	–	2.000	0.338, 11.846	0.445
10–12 times/year	–	–	–	–	–	–	2.154	0.349, 13.299	0.409
Above 12 times/year	–	–	–	–	–	–	1.528	0.213, 10.944	0.673
**Village doctor Intervention**									
Never	–	–	–	–	–	–	Ref	–	–
1–3 times/year	–	–	–	–	–	–	0.937	0.334, 2.625	0.901
4–6 times/year	–	–	–	–	–	–	0.343	0.076, 1.539	0.162
7–9 times/year	–	–	–	–	–	–	0.120	0.007, 2.098	0.146
10–12 times/year	–	–	–	–	–	–	0.058	0.005, 0.705	0.025^*^
Above 12 times/year	–	–	–	–	–	–	0.626	0.141, 2.792	0.540
**Stroke**									
**Pattern 1**									
Q1	Ref	–	–	Ref	–	–	Ref	–	–
Q2	6.721	1.346, 33.553	0.020^*^	19.711	2.636, 147.378	0.004^**^	61.906	4.332, 884.573	0.002^**^
Q3	5.952	1.214, 29.179	0.028^*^	12.38	1.777, 86.234	0.011^*^	53.385	4.104, 694.399	0.002^**^
Q4	3.223	0.616, 16.853	0.166	5.131	0.674, 39.035	0.114	21.235	1.430, 315.303	0.026^*^
**Village doctor Intervention**									
Never	–	–	–	–	–	–	Ref	–	–
1–3 times/year	–	–	–	–	–	–	2.384	0.616, 9.222	0.208
4–6 times/year	–	–	–	–	–	–	0.018	0.001, 0.335	0.007^**^
7–9 times/year	–	–	–	–	–	–	0.865	0.048, 15.717	0.922
10–12 times/year	–	–	–	–	–	–	0.000	0.000, 0.000	0.998
Above 12 times/year	–	–	–	–	–	–	0.130	0.012, 1.445	0.097
**Female**									
**Hypertension**									
**Pattern 1**									
Q1	Ref	–	–	Ref	–	–	Ref	–	–
Q2	1.632	0.872, 3.053	0.125	1.453	0.756, 2.794	0.263	1.395	0.706, 2.757	0.338
Q3	1.544	0.816, 2.92	0.182	1.568	0.793, 3.103	0.196	1.513	0.749, 3.056	0.248
Q4	3.012	1.365, 6.644	0.006^**^	3.203	1.349, 7.606	0.008^**^	2.933	1.209, 7.118	0.017^*^
**Diabetes**									
**Pattern 5**									
Q1	Ref	–	–	Ref	–	–	Ref	–	–
Q2	0.536	0.302, 0.949	0.032^*^	0.565	0.310, 1.027	0.061	0.626	0.339, 1.156	0.134
Q3	0.354	0.191, 0.653	0.001^**^	0.384	0.204, 0.724	0.003^**^	0.397	0.207, 0.761	0.005^**^
Q4	0.358	0.198, 0.648	0.001^**^	0.411	0.219, 0.770	0.006^**^	0.406	0.211, 0.783	0.007^**^
**Hyperlipidemia**									
**Pattern 3**									
Q1	Ref	–	–	Ref	–	–	Ref	–	–
Q2	1.574	0.800, 3.099	0.189	1.628	0.809, 3.274	0.172	1.585	0.766, 3.281	0.215
Q3	1.870	0.976, 3.584	0.059	2.205	1.120, 4.340	0.022^*^	2.254	1.121, 4.530	0.023^*^
Q4	1.217	0.605, 2.447	0.582	1.340	0.650, 2.763	0.428	1.347	0.634, 2.863	0.438
**Village doctor health education**									
Never	–	–	–	–	–	–	Ref	–	–
1–3 times/year	–	–	–	–	–	–	0.460	0.237, 0.891	0.021^*^
4–6 times/year	–	–	–	–	–	–	0.379	0.162, 0.887	0.025^*^
7–10 times/year	–	–	–	–	–	–	0.645	0.194, 2.149	0.475
Above 10 times/year	–	–	–	–	–	–	0.369	0.137, 0.989	0.048
**CHD**									
**Pattern 3**									
Q1	Ref	–	–	Ref	–	–	Ref	–	–
Q2	0.314	0.144, 0.685	0.004^**^	0.289	0.127, 0.658	0.003^**^	0.269	0.113, 0.638	0.003^**^
Q3	0.881	0.475, 1.633	0.688	0.816	0.415, 1.606	0.557	0.774	0.385, 1.556	0.472
Q4	0.480	0.233, 0.989	0.047^*^	0.499	0.231, 1.075	0.076	0.453	0.205, 1.002	0.051
**Village doctor intervention**									
Never	–	–	–	–	–	–	Ref	–	–
1–3 times/year	–	–	–	–	–	–	0.468	0.220, 0.997	0.049^*^
4–6 times/year	–	–	–	–	–	–	0.694	0.264, 1.824	0.459
7–9 times/year	–	–	–	–	–	–	0.325	0.031, 3.438	0.350
10–12 times/year	–	–	–	–	–	–	1.102	0.289, 4.21	0.887
Above 12 times/year	–	–	–	–	–	–	1.540	0.557, 4.26	0.406

*Model 1: adjusted for age, education, marital status, nation, body mass index (BMI), and income; Model 2: add community doctor follow-up, village doctor intervention and village doctor health education based on Model 1*.

*Q1 was the reference group*.

* *p < 0.05*,

** *p < 0.01*.

### Role of Health Management in the Link of Dietary Pattern and Chronic Diseases

In the present study, it was interesting that the relationship between dietary patterns and chronic diseases became stronger after adjusted health management (Figure 1). It could be seen that hyperlipidemia had a very close association between health management and chronic diseases. Moderate intervention (10–12 times/year: OR 0.185, 95% CI 0.039, 0.876) and appropriate health education (1–3 times/year: OR 0.553, 95% CI 0.321, 0.952; 4–6 times/year: OR 0.432, 95% CI 0.218, 0.856) in follow-up conducted by village doctor could significant reduce the risk of hyperlipidemia.

After stratified by sex further, the association between diabetes and pattern 5 disappeared when the factors of health management were added in the model, while health education became the independent protective factor for diabetes (4–6 times/year: OR 0.196, 95% CI 0.041, 0.953) in men, but not in women. In hierarchical analysis, health education provided by village doctors still benefited to reduce the prevalence of hyperlipidemia both in men and women. After adjusted health management, Q4 of pattern 5 (OR 4.398, 95% CI 1.235, 15.660) in men and Q3 of pattern 3 in women (OR 2.254, 95% CI 1.121, 4.530) were more likely to develop hyperlipidemia. All participants had profit to avoid CHD because of village doctor intervention. Community doctor follow-up (1–3 times/year) could reduce the prevalence of CHD (OR 0.320, 95% CI 0.115, 0.867) in men. What is more, moderate intervention (4–6 times/year) provided by village doctor could prevent the stroke in men (OR 0.018, 95% CI 0.001, 0.335).

### Multimorbidity Status

Multimorbidity in the present study refers to the coexistence of two or more chronic diseases in one patient (23). The prevalence of multimorbidity across sex categories is shown in Table 8. The results confirmed the close relationship between dietary consumption of pattern 5 and multimorbidity again in all subjects, especially in women as well. In addition, it was found that widower (OR 3.342, 95% CI 1.089, 10.316) was more likely to suffer from multimorbidity than single (Table 9), but this association was not significant in different sex. Health management also influenced multimorbidity status. Community doctor follow-up (1–3 times/year: OR 0.330, 95% CI 0.145, 0.753) and village doctor intervention (10–12 times/year: OR 0.110, 95% CI 0.015, 0.802) could reduce the risk of multimorbidity in men but not in women and overall participants.

**Table 8:** Association of prevalence of multimorbidity and dietary patterns across sex categories.

	**Multi-morbidity *n* (%)**	**Pattern 1**		**Pattern 2**		**Pattern 3**		**Pattern 4**		**Pattern 5**	
		**β**	***P***	**β**	***p***	**β**	***p***	**β**	***p***	**β**	***p***
Total (*N* = 697)	297 (42.6)	0.006	0.864	−0.012	0.740	−0.007	0.860	−0.037	0.323	−0.076	0.042^*^
Male (*N* = 241)	100 (41.5)	0.039	0.541	−0.025	0.697	−0.005	0.933	−0.036	0.569	−0.041	0.520
Female (*N* = 456)	197 (43.2)	0.002	0.966	0.004	0.929	−0.013	0.775	−0.036	0.442	−0.094	0.044^*^

* *p < 0.05*.

**Table 9:** Factors contributing to multimorbidity across sex categories.

	**Total**			**Male**			**Female**		
	**OR**	**95% CI**	***p***	**OR**	**95% CI**	***p***	**OR**	**95% CI**	***p***
**Marital status**									
Single	Ref	–	–	Ref	–	–	Ref	–	–
Married	2.039	0.725, 5.734	0.177	1.309	0.314, 5.454	0.712	2.982	0.625, 14.227	0.171
Separated/divorced	2.100	0.343, 12.858	0.422	5.437	0.379, 77.926	0.213	0.000	–	0.999
Widower	3.342	1.089, 10.316	0.036^*^	2.221	0.393, 12.565	0.367	4.833	0.936, 24.946	0.060
**Community doctor follow-up**									
Never	Ref	–	–	Ref	–	–	Ref	–	–
1–3 times/year	0.736	0.501, 1.082	0.119	0.330	0.145, 0.753	0.008^**^	0.936	0.576, 1.520	0.789
4–6 times/year	0.794	0.478, 1.318	0.372	1.120	0.411, 3.054	0.824	0.748	0.386, 1.452	0.392
7–9 times/year	0.508	0.204, 1.267	0.146	0.508	0.099, 2.593	0.415	0.655	0.198, 2.165	0.488
10–12 times/year	0.692	0.295, 1.625	0.398	2.404	0.472, 12.240	0.291	0.355	0.107, 1.170	0.089
Above 12 times/year	0.480	0.191, 1.203	0.117	0.374	0.050, 2.806	0.339	0.556	0.172, 1.798	0.327
**Village doctor intervention**									
Never	Ref	–	–	Ref	–	–	Ref	–	–
1–3 times/year	0.773	0.505, 1.183	0.236	1.066	0.454, 2.500	0.884	0.606	0.353, 1.041	0.070
4–6 times/year	1.167	0.680, 2.005	0.575	0.733	0.236, 2.278	0.591	1.150	0.578, 2.290	0.690
7–9 times/year	1.496	0.564, 3.967	0.419	0.772	0.137, 4.347	0.769	2.235	0.509, 9.818	0.287
10–12 times/year	0.667	0.285, 1.560	0.351	0.110	0.015, 0.802	0.029^*^	1.135	0.374, 3.446	0.823
Above 12 times/year	1.569	0.831, 2.964	0.165	1.915	0.507, 7.232	0.338	1.341	0.600, 2.996	0.474

* *p < 0.05*,

** *p < 0.01*.

## Discussion

Dietary pattern analysis, which might be especially valuable to the development and evaluation of food-based dietary guidelines, has emerged as an alternative and complementary approach for examining the association between diet and chronic diseases (5–7). Dietary pattern identification stresses the importance of holistic evaluation, indicating that different foods are consumed in intricacy combinations. Thereby, in evaluating the diet-disease relationship, the synergistic effect should be considered on health (9). In addition, health management, such as health education, intervention, and follow-up, by village or community doctor is very important daily work for chronic diseases prevention and control in Beijing nowadays. It is demonstrated to have a vital role in reducing the prevalence of chronic diseases. In this case, we explored the relationship between dietary patterns and chronic diseases among middle-aged and elderly in Huairou of Beijing, and the effect of health management was further detected.

First, we found that men and elder participants with lower education and obese people had higher incidence of chronic disease, which had been demonstrated in many countries (24–28). Women and obese people had a higher probability of suffering from hypertension. Diabetes was more prevalent in women. Young-old subjects and obese people were prone to have CHD, while population with higher education is opposite. In addition, women and young-old people were more likely to get a stroke. These results were similar to other research studies, which have shown that men, elder, low education, and high BMI played an important role in promoting chronic disease.

The comparison of dietary intake in different chronic diseases indicated that patients suffering from different diseases had the different dietary habits. People with hypertension had a high intake of vegetables and fruits. Patients who suffered from diabetes consumed more milk and less nuts, fruits, sugar, and alcohol. Patients with hyperlipidemia ate more seafood. Patients with CHD had less intake of meat and grease. These results of food intake were contrary to the conventional consideration of dietary risk factors (29, 30). In that, we focus on the relationship between dietary pattern and chronic diseases in the next step. From another point of view, this phenomenon might possibly prove that chronic disease patients have improved awareness of disease control and prevention.

In order to explore the synergistic effect of different kinds of foods, dietary patterns were derived by the factor analysis. Results definitely showed the correlation between dietary patterns and chronic diseases. There is evidence that intake of fat, sugar, or sugar-sweetened beverages was associated with high BMI, systolic blood pressure (SBP), and diastolic blood pressure (DBP), and a higher level of meat consumption is associated with lower SBP and DBP (31). In the present study, we observed that pattern 4, which is composed mainly of a large proportion of condiments and the highest PEF, was a risk factor for hypertension. The very high PEF resulting in hypertension is similar to the results of other research studies. The cooking oil use in rural people usually contains more saturated fatty acid that could influence blood pressure (32) and lead to chronic diseases (33, 34). These results reminded us that the high intake of grease and salt was still widespread in Huirou of Beijing countryside, and the consumption of sugar-sweetened beverages is also rising, which are important factors that lead to chronic diseases (35). However, from the perspective of energy intake, a lower energy intake of pattern 4 was a protective factor for diabetes. The different roles of pattern 4 in different chronic diseases suggested that various characteristics of the same dietary pattern may have different effects on specific chronic diseases (36).

Tubers as the staple food instead of refined grains can mitigate the postprandial glycemic excursion (37). This was proved in our result that pattern 5 was beneficial to diabetes as well. Meanwhile, the energy intake of pattern 5 was lower than the other patterns (the closest one to the dietary recommendation), which was demonstrated could reduce the prevalence of diabetes (38). Pattern 2 had a reasonable ratio of dietary energy source, which benefit to diabetes (39, 40). Results also indicated that pattern 3, which had the largest proportion of animal-food-derived protein, was a protective factor for CHD. Our finding was consistent with the report and meta-analyses which showed that dairy products' consumption did not adversely affect the risk of cardiovascular outcomes (cardiovascular disease, CHD, and stroke) and may make a contribution to reduce the prevalence of cardiovascular disease (41, 42). It might be related to the higher intake of high-quality protein (41).

Because we found chronic disease patients might have improved awareness on disease control and prevention, the characteristics of people who were prone to specific dietary patterns were tested. Men and people with higher income were positively associated with pattern 1, which had the highest energy and higher PEC consumption. Pattern 2, which had a proper energy supply ratio and more animal food, was more prevalent among men, middle-aged, lower BMI, and high-income population. While, pattern 3, as the high-quality protein sources, could be found much more in women, elder, higher education, and higher income subjects. These findings were consistent with several recent reports, which indicated that dietary patterns containing more healthy food were associated with a higher level of education, while people with lower socio-economic status might have poor-quality diet and less healthful dietary patterns (43–45). This reminded us that men, youth, lower education, higher BMI, and low income people might be the target population for receiving healthy nutrition education.

In order to further find out the role of health management in the relation between dietary patterns and chronic diseases, health management that includes health education, intervention, and follow-up by village doctor and community doctor were tested as interact factors. The most significant impact on hyperlipidemia could be seen in the result. Moderate intervention and appropriate health education could greatly subserve the prevention of hyperlipidemia. A recent study showed that education interventions have a meaningful positive impact on the effectiveness of chronic disease prevention in the Southeast Asian context, which is similar to the result (46). Some cohort study and intervention research also confirm that lifestyle intervention or integrated health education could contribute to controlling blood lipid levels in the normal range (47). The intervention also provides early support for patients to manage their lipids and prevent obesity through non-pharmaceutical interventions (48). According to the above results, dietary pattern with appropriate energy intake, source of energy supply, quality of macronutrients, and proper management of chronic diseases were significant for the chronic diseases prevention.

Moreover, hierarchical analysis by gender was detected as well. Results gave the evidence that the excessive level of energy intake may lead to strokes in men and hypertension in women. Besides the exploration of the significant impact on hyperlipidemia mentioned before, men were more likely to be affected by health education, intervention, and follow-up than women. These results reminded us that different aspects of dietary education and health management should be used in a different target population.

Finally, multimorbidity could be found nearly a half in our subjects. It was consistent with the evidence that multimorbidity was higher in elder people in Brazil (49). It also could be found that the prevalence of multimorbidity in women was higher than men, which was similar to the report of the China Health and Retirement Longitudinal Study (CHARLS) survey in 2015 (50). The multimorbidity also had a significance relationship with pattern 5, which indicated that the substitution of potato for grain could be a meaningful precaution. Well, men who suffered from multimorbidity were easier to accept health management. It hinted us again that enhancing the management of chronic diseases for men and finding other interventions for women, such as “expert patients,” are equally important (51).

This study had some limitations. The cross-sectional design could not draw conclusions about the etiological link among dietary patterns, health management, and chronic diseases, so it was impossible to describe causal relationships. Because of the limitation of subjects in the present study, the result was difficult to extrapolate to the general population. More research studies are needed to verify the conclusion.

## Conclusion

In conclusion, the association between dietary pattern and chronic diseases could be found in rural middle-aged and elder population in the countryside of Beijing. Health management plays important role in the link. The results of this study suggested that healthy dietary pattern combined with moderate management could decrease the risk of chronic diseases. A dietary pattern with appropriate energy intake, a reasonable source of energy supply, and high quality of macronutrients should be suggested. Long-term and interventional studies are needed to clarify the cause–effect relationship between dietary patterns, health management, and chronic diseases that can give suggestions to reduce the incidence of chronic metabolic diseases in middle-aged and elderly people in rural area.

## Data Availability Statement

The original contributions presented in the study are included in the article/supplementary materials, further inquiries can be directed to the corresponding author/s.

## Ethics Statement

Written informed consent was obtained from the individual(s) for the publication of any potentially identifiable images or data included in this article.

## Author Contributions

All authors listed have made a substantial, direct, and intellectual contribution to the work and approved it for publication.

## Funding

This work was supported by the National Natural Science Foundation of China (81973018 and 82003459). The Nutrition Research Fund of the Chinese Nutrition Society: Feihe Special Fund for Physical Nutrition and Health (CNS-Feihe2021-110).

## Conflict of Interest

The authors declare that the research was conducted in the absence of any commercial or financial relationships that could be construed as a potential conflict of interest.

## Publisher's Note

All claims expressed in this article are solely those of the authors and do not necessarily represent those of their affiliated organizations, or those of the publisher, the editors and the reviewers. Any product that may be evaluated in this article, or claim that may be made by its manufacturer, is not guaranteed or endorsed by the publisher.
